# Anisotropic Composition and Mechanical Behavior of a Natural Thin-Walled Composite: Eagle Feather Shaft

**DOI:** 10.3390/polym14020309

**Published:** 2022-01-13

**Authors:** Siyu Cai, Baoshuai Han, Yanjin Xu, Enyu Guo, Bin Sun, Yuansong Zeng, Hongliang Hou, Sujun Wu

**Affiliations:** 1 International Research Center for Advanced Structural and Biomaterials, School of Materials Science and Engineering, Beihang University, Beijing 100191, China; siyu_cai@buaa.edu.cn (S.C.); sunbin1995aa11@126.com (B.S.); 2 Avic Aviation Manufacturing Technology Research Institute, Beijing 100024, China; hbshit@126.com (B.H.); zengyuansong_avic@126.com (Y.Z.); hou_hl@163.com (H.H.); 3 Key Laboratory of Solidification Control and Digital Preparation Technology (Liaoning Province), School of Materials Science and Engineering, Dalian University of Technology, Dalian 116024, China; eyguo@dlut.edu.cn

**Keywords:** feather shaft, protein composition, tensile response, dynamic property, fracture behavior

## Abstract

Flight feather shafts are outstanding bioinspiration templates due to their unique light weight and their stiff and strong characteristics. As a thin wall of a natural composite beam, the keratinous cortex has evolved anisotropic features to support flight. Here, the anisotropic keratin composition, tensile response, dynamic properties of the cortex, and fracture behaviors of the shafts are clarified. The analysis of Fourier transform infrared (FTIR) spectra indicates that the protein composition of calamus cortex is almost homogeneous. In the middle and distal shafts (rachis), the content of the hydrogen bonds (HBs) and side-chain is the highest within the dorsal cortex and is consistently lower within the lateral wall. The tensile responses, including the properties and dominant damage pattern, are correlated with keratin composition and fiber orientation in the cortex. As for dynamic properties, the storage modulus and damping of the cortex are also anisotropic, corresponding to variation in protein composition and fibrous structure. The fracture behaviors of bent shafts include matrix breakage, fiber dissociation and fiber rupture on compressive dorsal cortex. To clarify, ‘real-time’ damage behaviors, and an integrated analysis between AE signals and fracture morphologies, are performed, indicating that calamus failure results from a straight buckling crack and final fiber rupture. Moreover, in the dorsal and lateral walls of rachis, the matrix breakage initially occurs, and then the propagation of the crack is restrained by ‘ligament-like’ fiber bundles and cross fiber, respectively. Subsequently, the further matrix breakage, interface dissociation and induced fiber rupture in the dorsal cortex result in the final failure.

## 1. Introduction

In recent years, fiber-reinforced plastic (FRP) has been proven to be light, stiff and strong [1]. Thus, FRP thin-walled structures have been viewed as exceptionally efficient components for aerospace and automotive engineering applications [2,3,4]. However, deciding on the best possible structural configuration still presents a challenge [5]. Intriguingly, a natural composite material, feather shaft, coincidently aligns with the goal to improve modern FRP thin-walled materials. Feather shafts stand out since they are extraordinarily lightweight, stiff and strong, yet able to flex, and have evolved for flight. A flight feather primarily bends and has to be aerodynamically affected during flight without destructive damage under flexural/torsional stresses. The feather shaft potentially exhibits a natural design strategy for thin-walled composite beams facilitating flight; it is of paramount importance to unravel the composition and mechanical behaviors of feather shafts.

**Figure 1:**
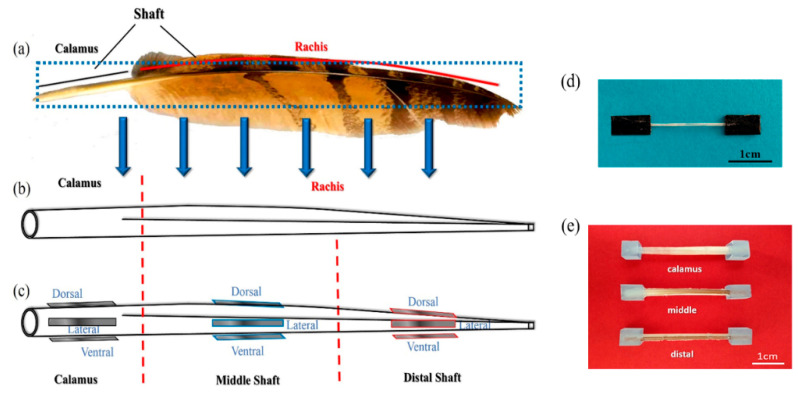
(**a**) The feather of eagle and (**b**,**c**) diagram of feather shaft and anisotropic cortex; the gray rectangles indicate (**d**) cortex samples located on different segments for tensile tests; (**e**) the shaft samples for bending tests.

The central shaft of a flight feather can be divided into two parts in length, called calamus (below the skin) and rachis (above the skin), as shown in Figure 1a. A whole shaft is composed of a keratinous sheath, enclosing a foamy core named the medulla; the cortex of the keratinous sheath contains the dorsal, lateral and ventral regions [6,7,8], as shown in Figure 1c. The feather cortex has been proven to be anisotropic along the length and circumferential directions of its shaft [9,10,11]. Previous research has reported that the feathers are entirely composed of β-keratin proteins [12,13,14,15,16], which generally show higher stiffness and strength than α-keratin-based materials [17]. Linghan and Murugan et al. [18] found the helical structure formed by β-keratin in the feathers and analyzed the significant effects of this structure on mechanical properties. Zou et. al. [19] further analyzed the compositions of the feather shafts by Fourier transform infrared (FTIR) spectrometer. However, there is still a lack of information about the detailed molecular compositions within cortex strips located at different positions on the feather shaft, considering their widely varied mechanical performances [9,10].

The feather shaft covered by the anisotropic cortex is significantly different from the conventional thin-walled structure. Wang and Meyers [10,11] reported that the variations of fiber arrangement depended on the specific cortex regions, including the axial, circumferential and crossed orientations. In addition, the quasi-static tensile tests on the cortex strips, which are excised from the dorsal, lateral and ventral regions, revealed that the Young’s modulus increased in the dorsal region towards the distal shaft, but consistently lower modulus in lateral cortex was exhibited, which corroborated the fibrous anisotropic structure [11]. However, no attempts have been made to incorporate the dynamic mechanical properties, e.g., storage modulus and damping, into a quantitative analysis for the anisotropic cortex strips of feather rachis, which, nevertheless, presents the real scenario for most flight feather shafts dealing with aerodynamic vibration frequently. The internal friction (i.e., damping), which relates to the conversion of vibration energy into internal energy during mechanical vibration, is regarded as a significant property for composites dealing with structural vibration. Moreover, it could also be used as a non-destructive testing method to study material microstructures due to the sensitivity to the variation of microstructure [20,21,22,23]. 

Widely present in nature and the engineering field, sandwich structures such as the feather shaft can efficiently make for resisting buckling and fracture during bending. As a cantilever beam with a thin-walled sandwich structure, the feather shaft bends both naturally (all feathers) and under aerodynamic forces (flight feathers). A few reports have described the response of the feather shaft in cantilever beam bending [24,25,26,27,28], three-point bending [29] and four-point bending [30], but there are few investigations of the corresponding damage and fracture behaviors of the shaft cortex during the bending process, which are significant to reveal the intrinsic mechanisms regarding the strengthening and toughening of the feather shaft to tackle the bending load elaborately.

In a bid to address the issues mentioned above, this work provides a systematic study of the anisotropic cortex of the eagle feather shaft, correlating to the features involving the design of bioinspired composites with novel thin-walled structures. In present work, the varied protein composition of the keratinous cortex located on different positions of the shaft is analyzed by Fourier transform infrared spectroscopy (FTIR). The dynamic properties of anisotropic cortex strips are investigated by a dynamic mechanical analyzer (DMA) using tensile vibration mode. The flexural behaviors and properties of feather shafts are tested by three-point bending tests, and a non-destructive technique for damage detection, acoustic emission (AE) [31,32,33,34], is simultaneously applied to monitor the real-time damage of specimens, further revealing the damage patterns and relevant fracture mechanisms of bent shafts. Our findings and analysis intend to reinforce our understanding of the eagle feather shaft and stimulate the design of novel synthetic structures that can reproduce the remarkable properties of the flight feather shaft.

## 2. Materials and Methods

### 2.1. Materials

Naturally shed covert in length of 21.3~24.7 cm of healthy adult eagle was purchased from a local zoo, under the Wildlife Permit. Feather shafts were obtained by cutting off the vanes for composition analysis and mechanical tests. The water content of natural feathers is typically lower than 10 wt.% [35,36], which conforms to the ambient-dried feathers [6]. Thus, the shafts were dried in vacuum at 105 °C for 2 h, and the mean moisture content were measured to be ~8.5 wt.%, similar to that of the natural feathers.

### 2.2. Analysis for Protein Composition of Cortex

The protein composition in cortex strips from different positions (dorsal, ventral and lateral files at calamus, middle and distal parts, respectively) of shaft sheath were characterized using an IRPrestige-21 Fourier transform infrared spectrophotometer (FTIR, IRPrestige-21, Shimadzu Corporation, Kyoto, Japan) over a wavenumber range of 400–4000 cm^−1^ under ATR mode. Thirty-two scans were accumulated for each spectrum at 25 °C.

### 2.3. Tensile Testing

Tensile tests were carried out to determine the tensile responses of dorsal, ventral and lateral cortex along the shaft length. Feather shafts were divided into three segments along the shaft length (i.e., calamus, middle shaft and distal shaft). The dorsal, lateral and ventral cortex strips along the shaft axis of each segment were excised (the medullary core is carefully removed to avoid scratches) to obtain thin rectangular samples. The length, width and thickness of each sample were measured with a vernier caliper. Tensile tests were performed following the ASTM D3039 [37], and the two ends of each rectangular strip were fixed with LOCTITE glue between two sand paper sheets, leaving a test gauge length of 20 ± 1.05 mm, as shown in Figure 1d. The width of the strip samples was 2.00 ± 0.11 mm, and the thickness varied from 0.10 ± 0.02 mm to 0.29 ± 0.03 mm. An Instron 5565 testing machine (Instron 5565, Instron, Boston, USA) equipped with 500 N load cell was used for tensile tests, and all specimens were loaded along the direction of feather shaft axis at room temperature with a strain rate of 0.01 mm/s.

### 2.4. Dynamic Mechanical Testing

The strip samples similar to tensile testing ones are used to measure the dynamic mechanical properties of anisotropic cortex. The tests were performed under tensile vibration at room temperature using a dynamic mechanical analyzer (DMA, Q800, TA Instruments, New Castle, DE, USA). The test gauge length is set as 10 mm, and the tests were performed at varied frequencies ranging from 1 to 50 Hz with the constant strain amplitude of 10 μm at room temperature and normal gas atmosphere.

### 2.5. Characterizations of Flexural Properties and Fracture Behaviors

The tubular samples were machined from the calamus, middle and distal parts along the shaft length, respectively, for three-point bending tests. Both ends of each tubular samples are embedded in epoxy to form a protective shell to prevent the samples twisting during testing. Loads are applied on the tubular samples, and care is taken to prevent compressing tubes and assure free rotation of the ends. Rubber pads on loading points and supporting bars are used to prevent local concentrated damage. Specimens ready for testing are shown in Figure 1e. According to the ASTM D790 [38], all specimens have a ratio of support length over depth (mean of five positions on shaft) of 16:1. The three-point bending tests were conducted using the Instron 5565 equipped with 500 N load cell, at a loading rate of 0.01 mm/s at room temperature for all specimens. Simultaneously, the fracture behaviors of samples were characterized through bending tests combined with acoustic emission (AE) system to monitor real-time damage process. The AE signals were recorded and analyzed by a digital signal processor with AEwin v2.19 AE system (AEwin v2.19, Physical Acoustic Corporation, Princeton Junction, USA).

## 3. Results and Discussion

### 3.1. Protein Composition of the Feather Shafts

**Figure 2:**
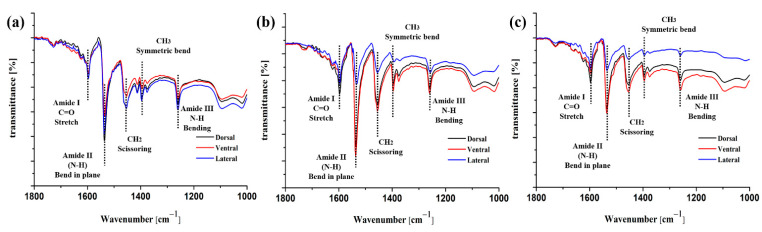
Infrared spectrum of the (**a**) calamus, (**b**) middle and (**c**) distal shaft.

The FTIR spectra for cortex from different segments and regions of feather shaft are shown in Figure 2, generally agreeing with that of typical keratin materials, and Amide I, II, and III features are identified, which are largely based on normal coordinate analysis pioneered by the notation in previous studies [39,40,41]. As structural repeat unit of protein, the peptide bond exhibits a number of IR-active amide bands [42,43], which is shown in Table 1. Characteristic peaks of amide I absorption stems from the C=O stretching vibration of the amide group, which gives rise to IR band(s) from the wavenumber range of 1600–1700 cm^−1^. Besides, the amide II bands originate from the N–H bending and are conformationally sensitive, and the amide III bands are very complex, resulting from mixtures of several coordinate displacements [42]. The absorption areas ~1468 cm^−1^ and ~1378 cm^−1^ are characteristic peaks corresponding to methylene (–CH_2_– scissoring) and methyl (–CH_3_ symmetric bend), respectively.

In Figure 2a, the FTIR spectra of dorsal, ventral and lateral cortex on calamus shaft are similar, indicating the uniformity of composition within calamus cortex. For these regions on rachis (Figure 2b,c), the peak intensity of C=O stretching (amide I) and N–H bending (amide II) of dorsal and ventral cortex are much higher than that of lateral cortex. Besides, the cortex on middle shaft exhibits higher C=O and N–H peak intensity than that of calamus and distal shaft (Figure 2b,c). Major factors responsible for conformation include hydrogen bonding and couplings between transition dipoles [43,44]. The C=O vibrational mode is closely related to the conformation for protein backbone and usually forms hydrogen bonds (HBs) by coupling to in-phase bending of the N–H bond. The wavenumber variations of C=O stretching (amide I) and N–H bending (amide II) indicate a different number of HBs between the amino acids [45]. Previous studies reported that introducing hydrogen bonding into main chains was an available method to significantly enhance the overall performance of protein, such as mechanical properties, elastic resilience, thermal properties, dimensional stability and so on [45,46,47,48,49]. It can be supposed that the larger amount of C=O stretching and N–H bending could give rise to the increase of HBs from lateral to dorsal and ventral cortex, thus possibly resulting in the enhancement of strength and stability of keratin within dorsal and ventral cortex. Similarly, the amount of HBs within cortex of middle shaft is deduced to be larger than that of calamus and distal shaft.

Additionally, the absorption areas ~1468 cm^−1^ and ~1378 cm^−1^ are characteristic peaks corresponding to methylene (–CH_2_– scissoring) and methyl (–CH_3_ symmetric bend), respectively [42]. As shown in Figure 2b,c, the intensity of –CH_2_– scissoring and –CH_3_ symmetric bend in dorsal and ventral cortex are much higher than that in lateral cortex, and the intensity of these conformations within middle shaft (Figure 2b) is generally greater than calamus (Figure 2a) and distal (Figure 2c) ones, whether in dorsal, ventral or lateral cortex. The researchers had found that specific branched chain structures, such as methylene, methyl, amino and phenyl in polymers, exhibited specific area of damping peak. The peak area is proportional to the number of these branched chain structures, indicating positive correlation between branched chain and dynamic viscoelastic properties of polymers, especially loss modulus and damping [50,51,52]. The increased volume and number of the side groups on the backbone can promote steric hindrance, leading to great inhibition for the segmental motion to enhance the mechanical loss of polymer. Therefore, it can be supposed that relatively higher content of –CH_2_– and –CH_3_ structures could improve the damping of dorsal and ventral cortex on rachis, and the damping of middle shaft cortex may be superior to proximal and distal shaft cortex. 

The distribution of characteristic peaks in FTIR spectra is consistent with previous studies that focus on investigating the protein composition of feathers [19,53]. Nevertheless, as shown in Figure 2, the widely varied peak intensity of characteristic peaks for different positions of feather shaft is firstly clarified in this work, which indicates the different content of C=O stretching, N–H bending and branched chain. The anisotropic distribution of protein configuration may influence the quasi-static and dynamic mechanical properties of different segments of shaft, which will be investigated below.

### 3.2. Tensile Response

For the purpose of determining the maximum tensile stress under which the DMA tests could be performed without breaking the cortex specimens, and assisting to clarify the damaged behaviors for different segments of feather shaft, the tensile responses of feather cortex are examined.

**Figure 3:**
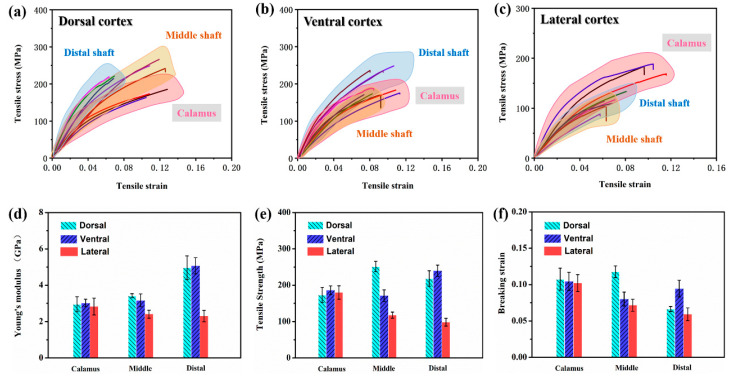
The tensile stress–strain curves of (**a**) dorsal, (**b**) ventral and (**c**) lateral cortex samples processed from calamus, middle and distal shaft, respectively; and the calculated (**d**) Young’s modulus, (**e**) flexural strength and (**f**) breaking strain of cortex samples (the error bar represents strand error for three samples).

The stress–strain curves of stretched cortex strips from dorsal, ventral and lateral regions along the shaft length are presented in Figure 3a–c, respectively. The Young’s modulus, tensile strength and breaking strain are calculated and shown in Figure 3d–f. It can be proved that the tensile properties of cortex strips are anisotropic. The elastic region can be observed initially in all curves, following a short and non-linear deformation region and then final failure. As shown in Figure 3d–f, the Young’s modulus and tensile strength of dorsal cortex are increased from the calamus to the distal shaft (40.53% and 20.69% amount of increase, respectively), while the breaking strain is increased and then decreased towards distal shaft. Similar to the dorsal cortex, the Young’s modulus of ventral cortex is also increased from calamus to distal shaft (39.94% increase), but the tensile strength and breaking strain are initially increased from calamus to middle shaft and then decreased towards distal shaft. Moreover, both the Young’s modulus and tensile strength of lateral walls are notably decreased from calamus to distal shaft (18.53% and 45.46% amount of decrease, respectively), and the breaking strain also decreases.

In addition, it should be noticed that at the calamus regions, the cortex from dorsal, ventral and lateral exhibits almost the same modulus (~2.95 GPa), strength (~180.23 MPa) and breaking strain (~0.11) (Figure 3d–f). The modulus and strength of dorsal and ventral cortex on middle shaft are much higher than lateral regions. Besides, the ventral cortex is relatively fragile, which may be due to a ventral groove being a weak point and thus being prone to split before fracture [10].

**Figure 4:**
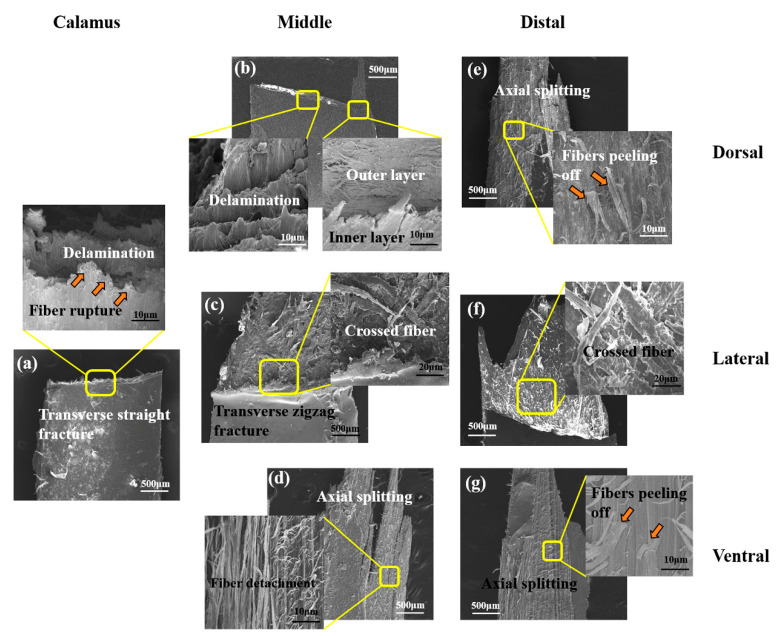
Typical fracture morphologies of stretched samples: (**a**) calamus cortex; (**b**–**d**) middle cortex through dorsal, lateral and ventral, respectively; (**e**–**g**) distal cortex through dorsal, lateral and ventral, respectively.

Correspondingly, the deformation and fracture mechanisms for stretched cortex also depend on the particular cortical regions and locations. As for calamus, all of the cortical specimens exhibit transverse straight fracture, and the delamination and rupture of majority axial fibers is dominant damage pattern of these samples (Figure 4a). It is due to the circumferential fibers holding the axial fibers in all of dorsal, ventral and lateral cortex on the calamus, which restrains the shear damage, inhibiting split of matrix between the axial fibers. This agrees with the homogeneous two-layered fibrous structure of flight feathers reported in previous studies by Wang et al. [10,11].

At the middle shaft, the dorsal cortex also exhibits transverse straight fracture, and detached circumferential fibrous layer could be observed (Figure 4b), whereas the ventral cortex exhibits axial splitting through the ventral groove and delamination (Figure 4d). In Figure 4d, the solely axial fibers within ventral cortex can be observed, which hardly prevent failure from longitudinal splitting. Both dorsal and ventral cortex show lots of axial fibers, which are aligned with the tensile direction and therefore strengthen the fibrous structure of cortex. This is consistent with previous reports that the volume of circumferential fiber is reduced towards the distal shaft [54,55], and the Young’s modulus increases [56]. The morphology of lateral cortex on middle shaft shows extensive delamination with transverse and zigzag fracture edge (Figure 4c), which is attributed to the particular effect of crossed fibers on deflecting crack path under tensile stress. As for lateral cortex, the decrease of strength and modulus may be due to the fact that the structure is varied from circumferential fibers enclosing axial fibers at the calamus to crossed-fibers at the rachis. The fibers are not aligned with the tensile direction, and therefore the strength and modulus are reduced. Besides, the lateral cortex is significantly thinner than the dorsal and ventral ones to be easily penetrated by cracks.

At the distal shaft, both dorsal and ventral cortex show major splitting fracture with a larger degree of axial detachment and fibers peeling off (Figure 4e,g). It is shown that the solely axial fiber structure can hardly resist split of matrix between fibers, leading to the mechanical property penalty. Additionally, the matrix containing relatively fewer hydrogen bonds is easily broken, which probably leads to the catastrophic split of matrix under shear stress. Similar to the middle shaft, the lateral cortex on distal shaft also exhibits an extensive delamination staggered fracture morphology, and the crack is deflected due to the crossed fibers (Figure 4f).

This is consistent with a lot of previous research, indicating that the calamus cortex consists of homogeneous two-layered fibrous structure with outer circumferential fiber and inner axial fiber. The outer circumferential fibers within dorsal and ventral cortex are gradually eliminated from calamus to distal shaft. As for lateral cortex, the fibrous structure varied from the two-layered fibrous structure within calamus to crossed fiber structure within rachis.

### 3.3. Frequency Scans by DMA

Frequency scans by DMA provide information on viscoelastic properties and damping characteristics of the keratinous cortex. Correspondingly, dynamic properties obtained from frequency scans are storage modulus (*E*′), loss modulus (*E*″) and *tanδ*. *E*′ is the elastic portion of the modulus and indicates how much energy is stored. *E*″ is the loss portion of the modulus, which indicates how much energy is dissipated. The *tanδ* is ratio of the two modulus values (*E*″/*E*′) and is a measure of the damping capacity of the material. This value indicates how effective the material loses energy to friction, heat or molecular rearrangements, and is of significant importance for materials coping with aerodynamic load, e.g., flight feather shaft. The damping (*Q*^−1^) can be calculated according to the following equation [57]:

(1) $$Q^{-1}=tan\delta =\frac{E^{\prime \prime }}{E^{\prime }}$$

where *δ* is the loss angle between applied stress and strain.

**Figure 5:**
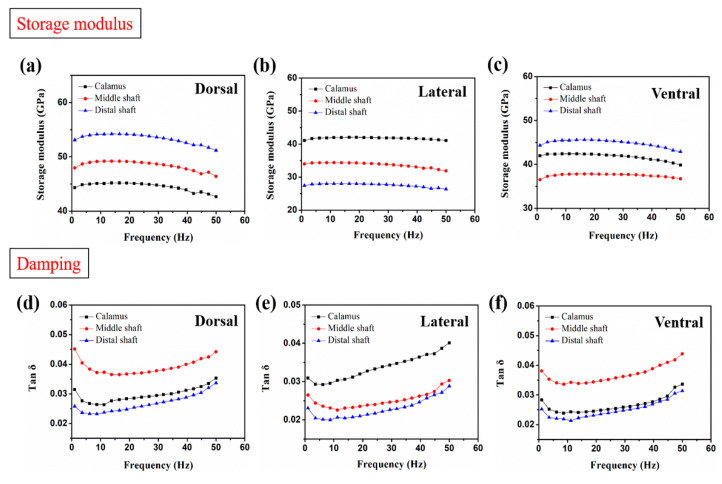
Plots of the storage modulus and damping of the (**a**,**d**) dorsal, (**b,e**) lateral and (**c**,**f**) ventral cortex, respectively.

As shown in Figure 5a–c, the storage modulus of the cortical samples is proved to be nearly constant from 1 to 50 Hz in testing frequency, indicating that the frequency has no obvious effect on the storage modulus of feather cortex. The DMA experimental results of storage modulus show similarly varied trends, with Young′s modulus calculated in Section 3.2. The results indicate that the storage modulus of dorsal cortex is increased from calamus to distal shaft (Figure 5a) and is gradually reduced in lateral cortex (Figure 5b). The previous papers have reported that deformability exhibits apparent variation according to the local fiber orientations; when the loading direction is parallel to the fibers, it forces them to be impeded by the matrix; loading perpendicular tends to separate fibers, and the force is mostly endured by the softer matrix [58]. Therefore, it can be assumed that the capacity of cortex to inhibit deformation and store of elastic deformation energy is enhanced as the increase of axial fibers in distal shaft, which leads to the improved storage modulus. Additionally, the damping of dorsal and ventral cortex at middle shaft is much higher than that at calamus and distal shaft (Figure 5d,f). However, the damping of lateral cortex both on the middle and distal shaft are approximate and are lower than that of calamus. It is consistent with the FTIR analysis in Section 3.1, which indicates the relatively higher content of branched chain within middle shaft than other segments, leading to the improvement of damping [50,51,52].

Furthermore, the dorsal, ventral and lateral regions of the calamus shaft all exhibit almost the same storage modulus and damping (Figure 5a–c). These results support the aforementioned analysis for calamus cortex—that the molecular compositions distributed in calamus cortex are mainly uniform, and the calamus cortex has a homogeneous fibrous structure, causing similar energy storage and dissipation capacity under dynamic loading. It is also notable that the damping of dorsal and ventral cortex on calamus shaft is close to that on distal shaft (Figure 5d,f), even though the amount of branched chain within the calamus cortex is proved to be higher than distal cortex. This compensation for damping performance may be caused by the effect of large number of interfaces between axial fibers and matrix on enhancing the internal friction, i.e., damping of composite [23]. The interfaces between the fibers and the matrix, which correspondingly distributed along the load direction, are easily inclined to slip and may be able to consume energy effectively.

### 3.4. Damage Behaviors of Bended Feather Shaft

**Figure 6:**
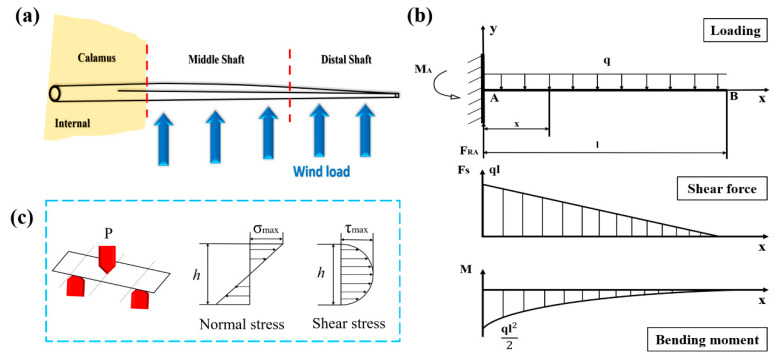
(**a**) The daily load pattern of the feather shaft, (**b**) schematic diagram for distribution of load, shear force and bending moment in feather shaft under wind load and (**c**) normal and shear stress distribution in flexural samples.

As shown in Figure 6a, the force pattern of flight feather shaft is similar to cantilever beam [30]. Accordingly, the distribution of load, shear force and bending moment through the outside rachis (including middle and distal shaft) are analyzed and shown in Figure 6b. Both the maximum shear force and bending moment are produced in middle shaft near the calamus and are gradually decreased towards the distal shaft. Thus, the integrated mechanical properties of middle shaft should be much better than other segments to tackle the complicated load states, which also conforms to the experimental results above.

**Figure 7:**
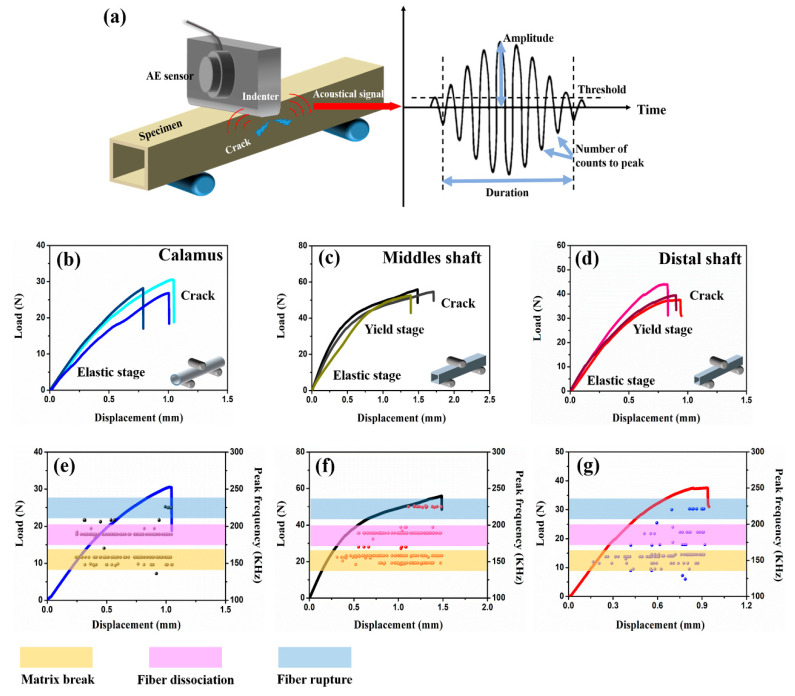
Experimental load-displacement behavior and real-time acoustic emission (AE) peak frequency signals of the calamus, middle shaft and distal shaft. (**a**) Illustration of the AE set-up and the signal characteristics. The load-displacement curves of (**b**) calamus, (**c**) middle shaft and (**d**) distal shaft under flexural load, respectively. Representative curves with related AE signals of (**e**) calamus, (**f**) middle shaft and (**g**) distal shaft under flexural load, respectively.

**Figure 8:**
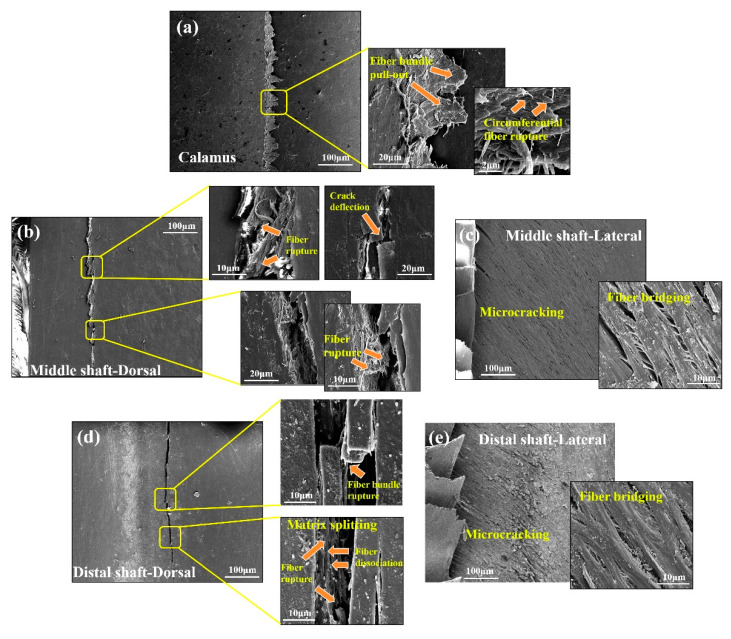
Fracture morphologies of (**a**) calamus, (**b**) dorsal cortex, and (**c**) lateral cortex of middle shaft, and (**d**) dorsal cortex and (**e**) lateral cortex of distal shaft.

The dorsal surfaces of specimens are loaded until the load dropped, which closely simulates the real stress condition of flight feathers [30]. The flexural stress–strain curves are shown in Figure 7b–d. The acoustic measurements are coupled with three-point bending tests to further research the mechanical behaviors of feather shaft, especially real-time damage patterns. The analysis for fracture morphology indicates three fracture modes at least: matrix breakage, fiber-matrix interface dissociation and fiber rupture (Figure 8). Similar to the results in previous works [32,33,34,59,60], the collected AE signals could be divided into high- (type C, from 220 to 250 kHz), medium- (type B, from 170 to 200 kHz) and low- (type A, from 130 to 160 kHz) frequency bands, corresponding to fiber rupture, fiber dissociation and matrix breakage, respectively.

The results for three-point bending tests on feather shaft indicate that the bending responses of calamus, middle and distal shaft are particularly different. The ultimate load of middle shaft is much higher than that of calamus and distal shaft, which benefits to adequately cope with severe force and large bending moment on middle shaft. The load-carrying capacity of calamus is relatively weak because it is hidden internally to avoid severe and complex load. Notably, the load-displacement curves indicate that calamus exhibits catastrophic failure after elastic stage, whereas both middle and distal shaft failed after elastic deformation and yield stage, and the yield stage of middle shaft is more durable than that of distal shaft.

The fibrous structure and corresponding variation of fracture patterns in different cortex regions is expected to influence the bending behaviors of shaft. As shown in Figure 8a, the failure mode of calamus is the same as the hollow and thin-walled composite tube, exhibiting straight buckling crack throughout the dorsal cortex under compressive stress. The zigzag edge containing debonding fiber bundles and broken circumferential fibers can be observed along the crack on calamus. The collected AE signals further clarify the damaged behaviors of calamus shaft, and both the type A (matrix breakage) and type B signals (fiber dissociation) are initially and simultaneously detected in elastic stage (Figure 7e), which indicates that the matrix breakage and fiber dissociation concurrent firstly. The continued type A and B signals are deemed to be associated with continuous damage in the matrix and interface after the crack initiation in matrix. The crack is propagated and then deflected by circumferential fibers, leading to the zigzag fracture morphology. While the load is increased, the type A and B signals persist, and the relatively concentrated type C signals are subsequently detected before final failure, implying that the fiber rupture gives rise to the final fracture of calamus.

However, only type A signals are initially detected during rachis bending, indicating prior occurrence of extensive matrix breakage. In rachis, large amount of discontinuous microcracks with bridging fibers can be observed in fracture morphologies of lateral cortex (Figure 8c,e). As mentioned in Section 3.1, the matrix in lateral cortex of rachis may be fragile due to the relatively few hydrogen-bond, which induces the prior fracture of the matrix under shear stress in lateral cortex (Figure 6c), causing large amount of type A signals in the first place. These disconnected microcracks dissipate the load energy, and the bridging crossed-fiber inhibits further growth of microcracks to avoid excessive propagation. The following type B signals occur near the origin of yield stage, implying that the crack propagates through the matrix and then penetrates the fiber-matrix interface to cause the yielding of shaft. As shown in Figure 8b, a lot of ‘ligament-like’ fiber bundles can be observed on dorsal cortex of middle shaft. The ‘ligament-like’ fiber bundles are laniated and exhibit irregular edge, which is supposed to result from the binding effect of circumferential fiber on crack propagation through the interface between axial fiber and matrix. However, the fracture morphology of distal shaft exhibits an axial smooth crack through the cortex, and fewer ‘ligament-like’ fiber bundles exist (Figure 8d), thus resulting in rapid failure of distal shaft after yield. The bending damage patterns of eagle feather shafts are similar to that of seagull, which also include deflected matrix crack, bridge ligament, ruptured fibers and unconnected microcracks [11]. These findings can facilitate clarification for strengthening and toughening mechanisms of these typical thin-walled biocomposites, e.g., the inhibition of crack propagation by bridging ligament to enhance structure and effect of microcracks on dissipating load energy to improve the toughness.

In summary, the combination analysis of AE signals and fracture morphologies can contribute to clarify the real-time damage behaviors of feather shaft under bending force. The anisotropic fibrous structure leads to different damage patterns of cortex in flexural feather shaft. 

## 4. Conclusions

The anisotropic features of the cortex are investigated, including the keratin composition, tensile response, dynamic mechanics properties and damage patterns of the thin-wall feather shaft. The significant findings of the current work are summarized as follows:

- The keratin composition of the calamus cortex is almost homogeneous. In rachis, both the HBs and the side-chain in the lateral cortex are less than that in the dorsal cortex and ventral cortex. Besides, the HBs and side-chain in the dorsal cortex of the middle shaft are much higher than that in other segments.
- The tensile properties, including Young’s modulus, tensile strength and breaking strain, are influenced by the keratin structure and fibrous structure. The dominant damage pattern of the stretched dorsal cortex varies from transverse fracture with fiber rupture to axial splitting towards the distal side of the shaft due to the gradual reduction of the circumferential fibers. The lateral cortex of rachis exhibits zigzag fracture with extensive delamination due to the crossed fibrous structure.
- The varied trend of the storage modulus of cortex strips is consistent with Young’s modulus. The storage modulus of the dorsal cortex on the distal shaft is the highest, and the lateral cortex shows lower storage modulus than other parts, which result from the different keratin composition and fibrous structure. The damping of dorsal and ventral cortex on distal shaft is relatively superior due to the larger amount of side chains and the interface motion between axial fiber and matrix.
- The shafts under bending load fail due to the fracture of dorsal cortex on compressive side. The matrix breakage and fiber dissociation firstly occur in calamus and then deteriorate; finally, the calamus fails due to the fiber rupture. Many ‘ligament-like’ fiber bundles can be observed on bended middle shaft, which are caused by initial matrix breakage and subsequent crack deflection by fiber. The distal shaft is broken by persistent matrix fracture with few fibers’ dissociation and rupture, leading to a smooth crack edge. Moreover, both in the middle and distal shafts, discontinuous microcracks are spawned on the lateral cortex and constrained by crossed fiber.
- Further study to design and fabricate a novel thin-walled structure, which is stimulated by findings and analyses, including the anisotropic features and damage behaviors of flight feather shaft in this work, is anticipated to be carried out in the future.

## Figures and Tables

**Table 1:** Band assignments of the main infrared active vibrations of keratin [41,42].

Wavelength Range (cm^−1^)	Functional Group
1600–1700	Amide I (mainly C=O stretch)
1480–1575	Amide II (N–H bend in plane)
~1468	CH_2_ scissoring
~1378	CH_3_ symmetric bend
1230–1330	Amide III (N–H bend in plane)

## Data Availability

The data presented in this study are available on request from the corresponding author.
